# The evolution of the endourologic management of pediatric stone disease

**DOI:** 10.4103/0970-1591.56177

**Published:** 2009

**Authors:** Marc C. Smaldone, Bishoy A. Gayed, Michael C. Ost

**Affiliations:** Division of Pediatric Urology, Children's Hospital of Pittsburgh, Department of Urology, University of Pittsburgh School of Medicine, Pittsburgh, Pennsylvania, USA

**Keywords:** Children, endourology, nephrolithiasis, percutaneous nephrolithotomy, shockwave lithotripsy, ureteroscopy

## Abstract

In the 1980s, the advent of shock wave lithotripsy (SWL) revolutionized pediatric stone management and is currently the procedure of choice in treating most upper tract calculi <1.5 cm in children. However, with miniaturization of instruments and refinement of surgical technique the management of pediatric stone disease has undergone a dramatic evolution over the past twenty years. In a growing number of centers, ureteroscopy (URS) is now being performed in cases that previously would have been treated with SWL or percutaneous nephrolithotomy (PCNL). PCNL has replaced open surgical techniques for the treatment of large stone burdens >2 cm with efficacy and complication rates similar to the adult population. Recent results of retrospective reviews of large single institution series demonstrate stone free and complication rates with URS comparable to PCNL and SWL but concerns remain with these techniques regarding renal development and damage to the pediatric urinary tract. Randomized controlled trials comparing the efficacy of SWL and URS for upper tract stone burden are needed to reach consensus regarding the most effective primary treatment modality in children. This report provides a comprehensive review of the literature evaluating the indications, techniques, complications, and efficacy of endourologic stone management in children.

## INTRODUCTION

Surgical management of urolithiasis in children has evolved dramatically over the past two decades. In the 1980s, the advent of shock wave lithotripsy (SWL) revolutionized pediatric stone management and is currently the procedure of choice in treating most upper tract calculi in industrialized nations. However, with miniaturization of equipment and refinement of technique, access to the entire pediatric urinary tract is now possible. In a growing number of centers, ureteroscopy (URS) is being performed in cases that previously would have been treated with SWL or percutaneous nephrolithotomy (PCNL). Recent data from large single institution series demonstrate stone free and complication rates with URS that are comparable to SWL but prospective trials are necessary before consensus regarding the most effective primary treatment modality can be reached. This report provides a directed review of the literature focusing on recent advances in the endourologic management of pediatric stone disease.

## INCIDENCE OF UROLITHIASIS IN CHILDREN

The incidence and characteristics of nephrolithiasis in children reflect a wide geographic variation, but stones occur in children of all ages without clear gender predominance. Although uncommon in the western hemisphere, pediatric stone disease is considered endemic in developing nations, including India, Turkey, Pakistan, and the far east. In these areas, ammonium acid urate and uric acid stones predominate, strongly implicating dietary factors.[1] Despite this discrepancy between hemispheres, nephrolithiasis in children is increasing in occurrence globally,[2] likely reflecting westernized lifestyle and dietary changes including higher salt intake with processed foods and decreased water consumption.

While children with anatomic abnormalities, urinary tract infections, and metabolic disturbances are considered to be at high risk for stone development,[3] evidence is accumulating that stones in a majority of westernized children are calcium based without any evidence of metabolic abnormality on 24-hour urine collection.[4] In a retrospective study of 1440 Pakistani children, Rivzi *et al*. reported that while diet, dehydration, and poor nutrition remain the major causative factors of pediatric nephrolithiasis, there is an emerging predominance over the past decade of upper tract calculi which is more consistent with adult populations.[1]

## DIAGNOSIS AND FOLLOW UP

Renal calculi in neonates and younger children are often diagnosed with ultrasound (US). While anatomic location and the associated presence of hydroureteronephrosis can be accurately assessed in a majority of children, up to 40% of calculi may be missed using US.[5] Despite the increased sensitivity of CT compared to US,[6] concerns regarding radiation exposure limit its use in young children. Although speculative, risk as high as one fatal cancer for every 1000 CT scans performed in young children has been reported.[7]

In our practice, asymptomatic calculi incidentally diagnosed with US are followed with serial US or plain abdominal films to minimize radiation exposure. Noncontrast helical CT is our diagnostic test of choice in older children presenting acutely with flank pain, but is reserved for younger children in which plain films or US are nondiagnostic. Following definitive therapy, children are ideally followed in a multidisciplinary stone clinic including urologic, renal, nutrition, and endocrine evaluation if necessary. Routine evaluation includes urine culture, 24-hour urine collection, and office-based US or abdominal films to detect recurrence. Follow up is individualized and based on age, anatomy, stone burden, and any underlying metabolic abnormality.

## CONSERVATIVE MANAGEMENT/CONSIDERATIONS IN CHILDREN

Conservative management of pediatric nephrolithiasis closely mirrors that of adults. Even in very young children, renal calculi <3 mm are likely to spontaneously pass, and stones ≥4 mm in the distal ureter are likely to require endourologic treatment.[8] In our practice, if a child's pain is controlled with oral analgesia, clear liquids are tolerated, and there is no evidence of urinary tract infection, parents are offered a closely monitored trial of spontaneous passage for 4–6 weeks prior to definitive therapy. Based on efficacy demonstrated in the adult population,[9] tamsulosin may be offered on an individualized basis as adjunctive therapy to facilitate ureteral expulsion. A ureteral stent is placed acutely in children with evidence of an infected genitourinary system, refractory colic, or uncontrolled nausea and vomiting. Under these circumstances definitive therapy is delayed 7–14 days following stenting to allow for system decompression, ureteral orifice dilation, and resolution of edema before endourologic management is undertaken.

Special considerations in the endourologic management of stone disease in children include preservation of renal development and function, prevention of radiation exposure, and minimizing need for retreatment. Despite advances in endourologic equipment and technique, controversy remains regarding the contribution of SWL to future development of diabetes or hypertension, and whether ureteral orifice dilation during URS leads to ureteral stricture formation or development of vesicoureteral reflux. International consensus is lacking as to the most effective surgical management of pediatric stone disease due to lack of prospective randomized trials comparing treatment modalities and disparity in the access to emerging technologies. Regardless of treatment modality, the presence of residual stone fragments (RFs) is associated with adverse clinical outcome,[10] and every attempt should be made to achieve a stone-free status. Surgeon experience is paramount to facilitate complete stone clearance and minimize retreatment rates. The decision regarding the most efficacious primary treatment modality must be individualized per child based on age, anatomy, location, and composition of stone burden.

## SHOCK WAVE LITHOTRIPSY

The emergence of SWL revolutionized the minimally invasive treatment of urolithiasis during the early 1980s. Initially reported in children in 1986,[11] large series have reported complication, safety, and stone-free rates comparable to adults[12–19] [Table 1]. When used as a primary treatment option for upper tract calculi, SWL efficacy ranges from 68–84%[12,15,20] and has become the preferred treatment modality for uncomplicated renal and proximal calculi ≤15 mm. Complications rates are minimal, and range in severity from hematuria and ecchymosis to obstruction with sepsis.[21] Although well tolerated in children, current stone-free rates with SWL are difficult to interpret from the existing body of data due to discrepancies between studies with regard to type of lithotriptor, number of shocks administered, and retreatment rates. Despite encouraging results, SWL has not been approved by the Food and Drug Administration for use in children, although it is a widely accepted treatment modality.

**Table 1:** Outcomes with large series of shock wave lithotripsy in children

Report	Children/renal units	Lithotripter	Mean age (yrs)	Stone location (%)	Mean size (mm)	Retreatment rate (%)	Stone free (%)	Complications (%)
Myers *et al*.[12]	446	Siemans	13.7 R	53.4 R	12.3 R	10.7 R	67.9 R	Sepsis - 0.2
		Lithostar	14.1 U	46.6 U	7.3 U	3.5 U	91.1 U	
Elsobkyetal. *et al*.[13]	148	Dornier MFL 5000 (106); Echolith MedTech (42)	11.2	92.6 R	10.2	64	86	Steinstrausse - 0.7
Muslumanoglu *etal.*[14]	344	Siemans	8.7	57.1 R	n/a	53.9	73.3	Overall - 9.6
		Lithostar Plus		42.9 U				Steinstrausse - 7.8
								UTI - 1.2
								Colic - 2.9
Rizvi *et al*.[15]	262	EDAP LT02	n/a	67.6 R	n/a	29.5	84.2 R	Colic - 10.1
		Technomed		32.4 U			54.1 U	Fever - 8.5
								Steinstrausse - 1.1
								Hematuria - 11.3
Ather *et al*.[16]	105	Dornier MPL 9000	5.6	100 R	15	n/a	95	Colic - 2.9
								Steinstrausse - 1.9
								UTI - 2.9
Aksoy *et al*.[17]	129/134	Dornier MPL 9000	8.7	84.4 R	15.7	n/a	85	Overall - 14.7
				15.6 U				Steinstrausse - 5.4
								UTI - 7.8
								Hematoma - .8
Razaetal. *et al*.[18]	122/140	Piezolith 2300; Dornier Compact Delta	7.7	n/a	17.9	n/a	69	Fever - 2.9
								Colic - 7.2
								Steinstrausse - 2.4
Demirkesen *et al*[19]	126/151	Siemans	8 (median)	66.9 R	10 R	40	71.5	Overall - 7.2
		Lithostar		33.1 LP	6 LP			Fever - 0.8
								Steinstrausse - 6.4

R – renal, U – ureteral, UP – upper pole, S – staghorn, LP – lower pole

### Shock wave lithotripsy technique in children

General anesthesia is administered in a majority of smaller children to avoid both patient and stone motion, and the need for repeated repositioning. With modern lithotriptors, intravenous sedation has been successfully employed in select older children.[22] Bowel preparation is seldom utilized to avoid dehydration and electrolyte imbalance postoperatively. The number of shocks delivered and the kilovoltage used vary per lithotriptor, but the current consensus is that low power settings (17–22 kV) be used to prevent stone migration during the procedure, with 3000 shock waves per session (<2000 in very young children).[21] Although still a controversial matter and dependent on stone burden and anatomy, we do not routinely stent children prior to SWL. However, ureteral catheters are occasionally employed to aid in the localization of radiolucent calculi.

### Stone size, location, composition, and patient age

While early series focused primarily on the feasibility, safety, and efficacy of SWL in children, recent efforts have centered on identifying demographic, anatomic, and stone related prognostic factors for treatment success. SWL is currently considered the primary treatment for upper tract calculi ≤15 mm in children,[21] but evidence supporting this stone size cut off is lacking, Ather *et al*. analyzed the correlation between stone size and clearance in 105 children younger than 14 years of age. They reported an overall stone-free rate of 95% after a mean of 1.7 treatments, with 5% of patients requiring additional procedures as adjuncts to SWL. With a maximum of 30 mm, mean stone size in the treatment-success group was 14 mm compared to 16 mm in the treatment-failure group.[16] In contrast, Elsobsky *et al*. reported a 91% stone-free rate versus 75% stone-free rate for mean stone diameter less than and greater than 10 mm, respectively.[13] While implicit that treatment of very large stone burdens with SWL would necessitate more shock treatments, more frequent retreatment sessions, and increased concern for postoperative obstruction, further study delineating a clear size cut off for uncomplicated upper tract stone burden is required to effectively counsel parents regarding the most effective first line therapy for renal calculi between 1 and 1.5 cm.

Renal anatomy and stone location has been subject of recent interest. The subject of frequent debate in the adult population, the most effective management of lower pole calculi in children has yet to be determined. Stone-free rates from initial small retrospective SWL series range from 56–61%[23,24] with retreatment rates as high as 40%.[24] SWL failure and retreatment rates were associated with increased mean stone burden,[24] increased infundibular length,[23] and infundibulopelvic angle greater than 45 degrees.[23] Staghorn calculi are uncommon in children and represent a management challenge. Although monotherapy success rates are low in adults, acceptable stone-free rates in children have been achieved with SWL. In 23 children stratified by age with a mean stone burden of 1.6 cm, Lottmann *et al*. reported an overall stone-free rate of 82.6% with only one case of symptomatic obstruction. A ureteral stent was placed in 22% of children, and these authors reported an 88% stone-free rate in children less than two years of age compared to 71% in children aged 6–11 years.[25] In 42 children with a mean stone burden of 3.2 cm stratified by ureteral stent placement, Al-Busaidy *et al*. reported an overall stone-free rate of 79%. While stent placement did not affect stone-free rates, they found that stent placement significantly reduced the major complication rate.[26] The superior success rates with SWL monotherapy in children compared to adults have been attributed to softer stone composition, smaller relative stone volume, increased ureteral compliance to accommodate stone fragments, and smaller body volume to facilitate shock transmission. SWL safety and efficacy have been demonstrated even in very young children. McLorie *et al*. treated 34 children younger than 3.5 years old (mean age 23 months) and reported an 86% overall stone-free rate (66% after one treatment) without major complications.[27] Treatment of proximal ureteral stones has achieved similar success rates to renal stones in most pediatric series, although ureteral stenting is more commonly employed to aid in stone localization and clearance.[12] Treatment of mid to distal ureteral calculi have historically been avoided in children due to difficulties with localization over the sacroiliac joint and concern regarding possible injury to developing reproductive systems.

Shock wave lithotripsy success by stone composition is similar between the adult and pediatric populations. Cystine stones are uniquely challenging due to their durability and high recurrence rates. While SWL monotherapy has demonstrated variable results in adults, there are few reports in the pediatric population. In a small recent series, Slavkovic *et al*. reported a 50% stone-free rate in six children with cystine stone burden ranging from.2–2.5 cm. Although stone-free rates were low, fragmentation was achieved in 100% of patients and the stone dissolution was achieved with medical therapy in the remaining children following SWL.[28] Authors have proposed that cystine stones formed within two years of therapy may be more easily fragmented with SWL and that stone number and not diameter may be more predictive of success.[21]

### Limitations and concerns

In children there is currently no consensus regarding the maximum size of RFs that are considered clinically significant,[3,21] and as a result there is no clear definition as to what constitutes ‘stone free’ status. While children have been shown to have a greater capacity to clear fragments than adults,[29] the presence of RFs has been correlated with adverse clinical outcome.[10] Afshar *et al*. followed 26 renal units with RFs ≤ 5 mm and reported that while 31% were asymptomatic with no fragment growth, 69% had adverse clinical outcomes including RF growth or clinical symptoms. Patients with RF had a significant increase in adverse clinical outcome compared to stone-free subjects, and the presence of metabolic disorders was associated with RF growth.[10] For these reasons, metabolic evaluations are now routinely being performed in children with history of calculi and every attempt should be made to achieve stone-free status. It is currently unclear if placement of a ureteral stent prior to SWL facilitates fragment passage and improves stone-free outcomes. Although prestenting rates are not consistent across series, current relative indications include cases of solitary kidneys, staghorn calculi, large ureteral calculi, obstruction, or abnormal anatomy and not based on total stone burden.

While SWL is well tolerated in children with few complications, stone-free rates following single session monotherapy remain as low as 44%.[14] As a result children are subjected to multiple treatments requiring general anesthesia.[22] The need for multiple treatment sessions is concerning since the effects of shock waves on renal tissue are unclear. A growing body of evidence in adults indicates that shock waves result in renal vessel vasoconstriction and that renal tubular injury and subcapsular hematoma from cavitation and shear forces are dependent on the kilovoltage applied.[30] In a large series of 340 adult patients with a mean follow up of 19 years post SWL, Krambeck *et al*. reported an increased risk of hypertension and diabetes mellitus related to bilateral treatment, number of administered shocks, and treatment intensity.[31] Although these results are concerning, differences between pediatric and adult populations and limitations inherent to a questionnaire-based retrospective study make application of these data in children difficult. Retrospective studies with limited follow up in children have reported that SWL and PCNL do not cause renal morphologic or functional alteration measured by GFR and serial DMSA functional studies,[32] but long-term data to date are unavailable. To eliminate confounding variables and fully address the risks of chronic renal damage from SWL long-term prospective data in children are clearly required.

## PERCUTANEOUS NEPHROLITHOTOMY

The safety and efficacy of PCNL for large stone burdens have been well established in adults. Initially urologists were reluctant to perform PCNL in children due to concerns regarding the use of large instruments in pediatric kidneys, parenchymal damage and the associated effects on renal function, radiation exposure with fluoroscopy, and the risks of major complications including sepsis and bleeding. However, with increasing experience[15,33–39] [Table 2], PCNL is currently being utilized as monotherapy and in combination with SWL (sandwich therapy) in children achieving stone-free rates ranging from 68–100%.[15,40] Although there is no current international consensus, relative indications for PCNL as primary therapy in children include large upper tract stone burden (>1.5 cm), lower pole calculi (>1 cm), concurrent anatomic abnormality impairing urinary drainage and stone clearance, or known cystine or struvite composition.[3,21]

**Table 2:** Outcomes with large series of percutaneous nephrolithotomy in children

Study	No. children/Renal units	Mean age (yrs)	Equipment	Stone size (mm)	Transfusion (%)	Stone free (%)	Sandwich therapy (%)	Complications (%)
Badawy *et al*.[33]	60	6	US	n/a	3.3	90	1.7	Fever - 8.3
								Colon injury - 1.7
								Urine leak - 3.3
								Open conversion - 5
Zeren *et al*.[34]	55/62	7.9	US, EHL	16.8	23.9	86.9	1.6	Fever - 29.8
								Open conversion - 1.6
Rizvi *et al*.[15]	62	n/a	US	47	25.3	67.7	27.4	Open conversion - 4.8
								Fever - 46.8
								Urine leak - 6.4
								Hydrothorax - 1.6
Desai *et al*.[35]	56	9.1	EHL	18.4	14.3	89.8	5.4	Urine leak - 5.4
Salah *et al*.[36]	135/138	8.9	US	22.5	0.7	98.6	0	Urine leak - 8
Holman *et al*.[37]	138	8.9	US	22.5	.4	98.5	0	Fever - 1.1
								Urine leak - 8
Samad *et al*.[38]	169/188	8.2	n/a	27.2	4	59.3	34.5	Fever - 42.8
								Hyponatremia - 0.1
								Obstruction - 0.1
Shokeir *et al*.[39]	75/82	6.6	US	14.4	1.2	95.1	4.8	Urine leak - 1.2

EHL – electrohydraulic lithotripsy; US – ultrasound, HL – holmium laser

Initially described in children by Mor *et al*. in 1997,[41] early pediatric PCNL series described the use of adult-sized instruments. Although initial series avoided performing PCNL in very small children (<5 years of age) due to concerns regarding parenchymal damage, multiple series utilizing adult-sized instruments have reported high efficacy rates with acceptable complication rates even when dilating tract size as high as 30 Fr.[34,36,38,42] Despite these successes, early efforts focused on developing technology to minimize percutaneous tract size without affecting PCNL efficacy. Jackman *et al*. developed a novel percutaneous access technique (‘mini-perc’) using a 13 Fr peel-away vascular access sheath and reported an 85% stone-free rate for 11 procedures in seven children with a mean age of 3.4 years.[43] The benefits of minimal tract dilation include increased maneuverability, decreased blood loss, and shorter hospital stay. However, theoretical limitations including prolonged operative times and impaired visualization from bleeding imply that this technique may not be adequate for very large stone burdens. Recent advancements in instrumentation such as smaller nephroscopes (15–18 Fr) and more efficient energy sources for intracorporeal lithotripsy including holmium:YAG laser and smaller pneumatic lithoclast and US probes have greatly facilitated percutaneous treatment techniques. As a result, PCNL has now replaced open surgery as the treatment of choice for large stone burdens in children of all ages.

### Percutaneous Nephrolithotomy Technique in Children

All percutaneous procedures are performed using general anesthesia and antibiotic prophylaxis. A warm operating room, body temperature isotonic irrigation solution, brief anesthetic induction, short operative times, proper draping, and monitoring of body temperature should decrease the incidence of hypothermia and hyponatremia. After induction of anesthesia with patient in the lithotomy position, a retrograde pyelogram is performed to outline the collecting system and an occlusive balloon catheter is left *in situ*. The patient is then repositioned in the prone position with the torso elevated at 30° from the table surface with a towel roll.[21]

After selection of the desired calyx, an 18 gauge spinal needle is placed with the assistance of fluoroscopy in two planes. The ideal tract is one that provides the shortest and most direct access to the stone. For complex calculi occupying multiple calices including the lower pole, a supraposterior access is preferred to provide visualization of the superior calyx and pelvis, access to the pelvis and ureter, and straight access to the inferior calices allowing easier manipulation of the working instruments and minimizing torque on the collecting system. Following initial puncture, no attempt should be made to redirect the needle while it is located within the cortex of the kidney to avoid trauma. After access is confirmed with urine or irrigation return, a flexible guidewire is placed into the collecting system through the needle and directed down the ureter into the bladder. A small skin incision is made with a No. 11 scalpel and 8 and 10 Fr coaxial dilators are passed over the guidewire into the collecting system. Once in place, an Amplatz Super Stiff™ guidewire is placed as a working wire.

Tract dilation can be performed by several techniques. Serial dilation with Amplatz dilators over working wires and subsequent sheath placement under fluoroscopic guidance is the most common technique employed. Alternatively, a 13 Fr peel-away sheath (Docimo Mini-Perc, Cook Urological Inc., Spencer, Indiana) and trocar are passed over the wire into the calyx under fluoroscopic guidance and the trocar is removed. We have had success using the NephroMax™ High Pressure Nephrostomy Balloon Catheter (Boston Scientific, Natick, Massachusetts) which facilitates dilation to 30 Fr at a pressure of 17 atmospheres. This technique permits dilation and sheath placement in a single step, thereby minimizing potential parenchymal trauma and bleeding from sequential dilation with metal dilators. While the decision to proceed with mini-perc or dilation is individualized based on child's age, anatomy, and stone burden, familiarity with all of the above techniques facilitates complete access with minimal morbidity.

Once access is obtained, nephroscopy and nephrolithotomy can be performed with a variety of energy sources for stone fragmentation. The outer diameter of nephroscopes range from 17–26 Fr and a 15 Fr flexible nephroscope with a 6 Fr working channel has also been developed. In addition, 7 and 8 Fr offset cystoscopes with 5 Fr working ports and 7–9 Fr flexible ureteroscopes can be used through an 11 Fr access sheath with enough clearance to allow low pressure irrigation.[3] Energy sources currently utilized include ultrasonic lithotripsy, electrohydraulic lithotripsy (EHL), and the holmium laser, although individual preference is determined by availability and surgeon experience. Postoperative stenting and/or placement of a nephrostomy tube are both patient and surgeon dependent and vary between series.

### Percutaneous nephrolithotomy outcomes

Recent large retrospective series of PCNL monotherapy have demonstrated high efficacy rates approaching 90%.[34,35,42] In 56 children (mean age 9.1 years) with a mean stone burden of 337.5 mm^2^, Desai *et al*. reported a stone-free rate of 89.8% utilizing EHL through a 14 Fr nephroscope and a 20–24 Fr sheath. Of these, 61% required multiple tracts and 45% were staged procedures. Findings demonstrated that the number and size of tracts were significantly associated with postoperative hemoglobin decrease (mean 1.9 g/dL) and overall transfusion rate (14%).[35] In 52 children with a mean age of 7.9 years and a mean stone burden of 282 mm^2^, Zeren *et al*. reported a 87% stone-free rate using US and EHL for fragmentation and tract dilation from 18–30 Fr. Complications included postoperative fever (30%) and need for transfusion (24%). Transfusion was associated with operative time, sheath size, and stone burden.[34] In 135 children aged 8.9 years with a mean stone burden of 507 mm^2^, Salah *et al*. reported a 98.5% stone-free rate utilizing US through a 26 Fr nephroscope. Complications were low (8% urine leak rate and 0.7% transfusion rate), with only one patient requiring a second procedure.[36] In a recent series of 46 children with a mean stone burden of 332 mm^2^, Bilen *et al*. reported a 88% stone-free rate using EHL, US, and the holmium laser. When stratified by tract size (14, 20, and 24 Fr), efficacy rates were similar in all groups, but there were no complications or transfusions in the 14 Fr tract group.[42]

In an effort to reduce the number of tracts and associated morbidity, some centers have chosen to follow primary PCNL with adjunctive SWL therapy to clear RFs. In a small series of 29 children with a mean age of 3.8 years and a mean stone burden of 2.4 cm, Mahmud *et al*. reported a 60% stone-free rate after PCNL monotherapy using EHL through a 17 Fr angled nephroscope. Only one tract was used in all patients, and after SWL sandwich therapy the stone-free rate increased to 100%.[40] In a larger series of 169 children with a mean stone burden of 3.1 cm, Samad *et al*. reported a 59% monotherapy stone-free rate with 96% of cases performed through a single tract. Approximately one-third (34.5%) of primary failures were treated with SWL; the cumulative stone-free rate in all patients was 93.8% with a 3.6% transfusion rate.[38] When stratified by age, anatomy, bilaterality, and renal function, stone-free outcomes were equivalent in all groups. The decision to follow PCNL with SWL is related to operator experience with percutaneous technique and available technology. It is our preference to perform a second look nephroscopy through the original tract to ensure stone-free status during the initial hospital admission rather than progress to SWL sandwich therapy. With continued improvement in technology and technique, the indications for PCNL in children will continue to increase. Technically challenging in nature, surgeon experience with PCNL is paramount in developing individualized treatment plans to optimize efficacy with minimal morbidity.

## URETEROSCOPY

Adoption of URS for the treatment of pediatric stone disease has lagged behind that of adults due to concerns regarding use of large ureteroscopes in small caliber ureters, a higher post SWL stone fragment clearance rate compared to adults,[29] and the low incidence of stone formation in children. Since the mid 1980s, with the acceptance of SWL as primary therapy for upper tract calculi <1.5 cm, URS has been historically utilized for calculi below the iliac crests, and for upper tract calculi after SWL failure.[3] URS was not considered primary therapy for upper tract stones in children due to concern for ureteral ischemia, perforation, stricture formation, and development of vesicoureteral reflux as a result of dilatation of small caliber ureteral orifices.

With significant improvements in both the miniaturization and durability of endoscopic equipment and the acceptance of the holmium laser, URS has become a more attractive option in young children. First described by Ritchey *et al*. in 1988,[44] early series utilizing rigid URS for distal ureteral stones reported stone-free rates ranging from 86–100% with low complication rates.[8,15,45–48] In a comparison of 31 children randomized to URS or SWL as primary therapy for distal ureteral stones, De Dominicis reported a significantly higher stone-free rate after one treatment (94 versus 43%) for children treated with URS.[46] The results of these retrospective studies have begun to refute the notion that dilation of the pediatric ureter will result in vesicoureteral reflux or the development of ureteral strictures. In a systematic review of the literature encompassing 221 pediatric ureteroscopies, Schuster *et al*. noted only two ureteral strictures and a minimal incidence of vesicoureteral reflux.[45]

Early successes with treatment of distal calculi in children[49–51] have led to a number of centers expanding its utility to the treatment of upper tract calculi [Table 3]. Findings from the first series treating upper tract calculi have recently become available, and demostrate stone-free rates between 88 and 100% with complication rates similar to that of the adult population.[48,52–55] Lesani *et al*. reported their experience using 4.5, 6, and 8 Fr rigid URS in treating proximal ureteral stones in 24 children with a mean age of 10.7 years. They did not perform ureteral dilation in any cases, and 100% of children were rendered stone free.[52] In a large series of 100 children with a mean stone diameter of 8.3 mm, of which 52% children had upper tract calculi, Smaldone *et al*. reported a 91% stone-free rate with 9% of children undergoing staged procedures. With a mean follow up of 10 months, they reported a 4.2% perforation rate managed with ureteral stenting and one distal ureteral stricture requiring open neocystostomy.[53] Corcoran *et al*. reviewed their cohort of 47 children (mean age 9.4 years) with upper tract calculi managed with flexible URS and holmium laser lithotripsy. With a mean stone burden of 10.2 mm, they reported an 88% stone-free rate with 26% requiring staged procedures.[54] Adoption of techniques utilized in the adult population, most notably sequential coaxial and balloon dilation of the ureteral orifice and use of ureteral access sheaths, has facilitated access to the entire pediatric urinary tract. Initially described in eight children by Singh *et al*.,[56] ureteral access sheaths have been shown to facilitate repetitive upper tract access, reduce intrarenal pressures, decrease operative time, and improve stone-free rates in adults. In our experience, use of ureteral access sheaths and the 6.9 Fr flexible ureteroscope has made possible treatment of lower pole calculi that would have previously required SWL or PCNL. Cannon *et al*. reported a 76% stone-free rate in 21 children with lower pole calculi and a mean stone diameter of 12.2 cm. After a mean of 11.4 months, no major complications were observed.[55] With the transition from SWL to URS as a primary treatment modality at our institution, current relative contraindications to ureteroscopic management include staghorn stones in recurrent stone formers more amenable to PCNL, anatomic anomalies making retrograde access difficult, and previous endoscopic failure.

**Table 3:** Outcomes with large series of rigid and flexible ureteroscopy in children

Study	No. children/ No. procedures	Mean age (years)	Stone size (mm)	Stone location (%)	Fragmentation	Ureteral orifice dilation (%)	Stone free (%)	Staged (%)	Post operative stenting (%)	Complications (%)
Rigid ureterosscopy for mid to distal ureteral calculi										
Al-Busaidy *et al*.[49]	43/47	6.2	12.6	100 U	EHL	n/a	93	n/a	n/a	Ureteral perforation - 4
										Ureteral stricture - 2
										Fever/VUR - 12
Bassiri *et al*.[50]	66/66	9	8	100 U	USL, EHL, PDL	37.9	88	n/a	n/a	Renal colic - 1.5
										Gross hematuria - 16.7
										Pyelonephritis - 4.5
Raza *et al*.[51]	35/52	5.9	9.4	100 U	PDL, EHL, HL	3.9	79.3	28.6	n/a	Ureteral stricture - 2
										Fever - 10
										Ureteral perforation - 6
Rigid and flexible ureterosccopy for upper tract and renal calculi										
Minevich *et al*.[48]	58/65	7.5	n/a	64.6 U 35.4 P	EHL, HL	30	98	n/a	85	Ureteral stricture - 1.3
Smaldone *et al*.[53]	100/115	13.2	8.3	52 R 48 U	HL	70	91	9	75	Ureteral perforation - 4.2
										Ureteral stricture - 1
Cannon *et al*.[55]	21/21	15.1	12.2	100 LP	HL	81	76	14	71	0
Corcoran *et al*.[54]	47/61	9.4	10.2	100 R	HL	91	88	26	70	Ureteral perforation - 9

EHL – electrohydraulic lithotripsy; HL – holmium laser, R – renal, U- ureteral, P – proximal, LP – lower pole

### Ureteroscopic technique in children

All ureteroscopic procedures are performed under general anesthesia to prevent patient movement and minimize the risk of ureteral perforation. Following antibiotic prophylaxis, patients are placed in the lithotomy position and rigid cystoscopy (7.5, 11, or 18 Fr) is performed to place a safety or working wire. Under fluoroscopic guidance, the guidewire is advanced into the renal pelvis or beyond the level of the stone. Ureteral orifice dilation is performed with 8/10 Fr coaxial dilators in ureters that have not been prestented, or when the rigid/flexible ureteroscope cannot easily be advanced. We generally do not use balloon dilation of the ureteral orifice due to concern for development of ureteral stricture from ischemia. Our bias is that use of the 8/10 dilator allows for tactile feedback regarding the tightness of the ureter, which is not available with balloon dilation. If we encounter difficulty with the 8/10 dilator, our preference is to place a stent and return for a second procedure rather than dilate more aggressively.

The decision to use a flexible (6.9 Fr) or semirigid (7.5 Fr) ureteroscope is made depending on size and location of stone, anatomic factors, and individual surgeon preference. A 4.5 Fr rigid ureteroscope is also currently available for use although our experience with this device is limited. Rigid or semirigid URS is routinely performed with a safety wire in place while flexible URS is performed with both a safety and working wire in place. Ureteral access sheaths (internal diameter of 9.5 or 12 Fr) are utilized in select cases to facilitate flexible URS in cases of large proximal ureteral or renal pelvis stone burdens. Body temperature irrigating fluid, which may be used under pressure, should be isotonic to avoid hypothermia and hyponatremia. Calculi are extracted with a basket when feasible or fragmented using the holmium:YAG laser to facilitate removal. Other energy sources for fragmentation available include US lithotripsy and EHL. The decision to place a ureteral stent postoperatively is made based on the duration of the procedure, number of passes with the ureteroscope, and degree of visible ureteral trauma or edema at the conclusion of the procedure. If the patient can tolerate leaving a urethral string in place for three days to one week, the patient's parents are asked to remove the stent at home, otherwise the stent is removed under brief anesthetic after seven days.

### Concerns and limitations

With smaller, more durable ureteroscopes and improved optics for visualization, URS is becoming more prominent in the pediatric endourologists armamentarium of stone management techniques. However, many unanswered questions still need to be addressed. In postpubertal children with an adult body mass, ureteroscopic access is technically similar to the adult population. In the small prepubertal child, questions regarding whether to attempt primary treatment without ureteral orifice dilation, perform dilation at the time of definitive therapy, or to place a stent and allow the ureter to passively dilate prior to definitive therapy remain unanswered. In 29 children with a mean age of 11 years, Herndon *et al*. performed semirigid URS with 4.5 and 6.5 Fr ureteroscopes for distal ureteral calculi. Fourteen percent of children were prestented, but no child was actively dilated. The ureter was accessed in 100% of cases for a stone-free rate of 96%.[57] Since our flexible and semirigid ureteroscopes are 6.9 and 7.5 Fr, respectively, it is our preference to sequentially dilate with the 8/10 coaxial dilator even in very young children, but if we encounter difficulty a stent is placed rather than dilate more aggressively. While we feel this approach minimizes long-term complication rates, particularly in the management of upper tract calculi, it increases the number of children that require a second anesthetic and procedure to achieve stone-free status. Our recent finding that 40% of pediatric patients will require at least two procedures to treat upper tract calculi ureteroscopically suggests that with the current equipment, the likelihood of achieving a stone-free state after one ureteroscopic procedure may not be significantly better than with SWL.[54] Another area of contention is the necessity of placing a post URS stent in all children. While the tendency in large series has been to leave a stent in place after ureteroscopic manipulation in a majority of children,[53] some authors have reported no acute or long-term sequelae despite leaving a post URS stent in less than 20% of cases.[54] In our experience, the decision to place a post URS stent is made on an individual patient basis and is dependent on surgeon experience and degree of visible ureteral trauma at the conclusion of the procedure.

## LAPAROSCOPIC AND ROBOTIC-ASSISTED PYELOLITHOTOMY

Treatment of large stone burdens in children is technically challenging often requiring multiple procedures. Laparoscopy and robotic-assisted laparoscopy have been utilized successfully in adults for treatment of calculi during the concomitant treatment of ureteropelvic junction obstruction and in the primary treatment of staghorn calculi. Small series utilizing these techniques in children have only recently been described. In eight children (mean age, four years) with a mean stone burden of 2.9 cm undergoing transperitoneal laparoscopic pyelolithotomy, Casale *et al*. reported a 100% success rate, a mean hospital stay of 2.15 days, and a mean operative time of 1.6 hours with no major complications.[58] In the first report of robotic-assisted laparoscopic pyelolithotomy, Lee *et al*. described their experience in five patients; four with cystine staghorn calculi refractory to PCNL and SWL and one with calcium oxalate calculi and concurrent ureteropelvic junction obstruction. Of these cases, four were completed robotically, with one patient having a residual 6 mm lower pole stone and one patient required conversion to an open procedure. Mean operative time in this series was 315 minutes, mean estimated blood loss was <20 cc, and the mean hospital length of stay was 3.8 days.[59] These early experiences demonstrate that laparoscopic pyelolithotomy is feasible, safe, and efficacious as an alternative to open pyelolithotomy in children and warrants further study. However, due to their demanding technical nature, these procedures will likely be limited to endourologic management failures in academic centers with abundant expertise in laparoscopic and robotic pediatric surgery.

## DETERMINATION OF STONE-FREE STATUS

As the surgical management of pediatric stone disease evolves, the lack of a consistent definition of ‘stone free’ following definitive therapy is an issue that remains unaddressed. Although controversial, in select adult patients all stone fragments can be considered clinically significant and can lead to stone recurrence.[60] Likewise, the presence of RFs in children has been associated with poor outcomes,[10] and any size stone fragment in a young stone former may result in the need for repeat surgical procedures. However, these fragments often are not detected on US or KUB necessitating reliance on CT imaging in select children.

Balancing the risks of radiation exposure for post-treatment stone detection and the risks of anesthesia for secondary procedures is a challenging dilemma for contemporary pediatric endourologists. Newer, high speed helical CT scanners reduce radiation exposure and rarely require intravenous sedation. In addition, maximizing intraoperative fragment detection by direct visualization in URS and PCNL and continued development of high resolution real-time fluoroscopy may result in less reliance on postoperative imaging and decrease the need for second look nephroscopy/URS, SWL, or sandwich therapy.[61] Until the risks of radiation exposure in children are more clearly defined, surveillance in these children will be individualized based on age, anatomy, stone burden, and underlying metabolic abnormalities.

## CONCLUSIONS

Evolution of technique and miniaturization of instruments have changed the management of pediatric stone disease. However, despite encouraging results, concern remains regarding safety of endourologic treatment in smaller patients and its subsequent effects on the growing kidney. While SWL is still considered first line therapy for upper tract calculi <1.5 cm, there is increasing evidence that SWL and URS are equally safe and efficacious for upper tract stone disease in children. While PCNL remains the most effective technique for large upper tract stone burdens, there are recent reports of laparoscopic and robotic-assisted laparoscopic pyelolithotomy in major pediatric academic centers with extensive laparoscopic and robotic experience. With no prospective randomized evidence currently available, individual surgeon experience is the most important determining factor in counseling patients regarding the most effective primary treatment option. Similar to endourologic management in the adult population, familiarity with the full spectrum of endourologic techniques facilitates a minimally invasive approach to the entire pediatric urinary tract.
